# Therapeutic effect of budesonide/formoterol, montelukast and N-acetylcysteine for bronchiolitis obliterans syndrome after hematopoietic stem cell transplantation

**DOI:** 10.1186/s12931-016-0380-1

**Published:** 2016-05-26

**Authors:** Sei Won Kim, Chin Kook Rhee, Yoo Jin Kim, Seok Lee, Hee Je Kim, Jong Wook Lee

**Affiliations:** Division of Pulmonary, Allergy and Critical Care Medicine, Department of Internal Medicine, College of Medicine, The Catholic University of Korea, Seoul, Korea; Division of Hematology, Department of Internal Medicine, College of Medicine, The Catholic University of Korea, Seoul, Korea

**Keywords:** Bronchiolitis obliterans syndrome, Inhaled corticosteroid, Long-acting β_2_-adrenergic agonist, Montelukast, n-acetylcysteine

## Abstract

**Background:**

Bronchiolitis obliterans syndrome (BOS) after allogeneic hematopoietic stem cell transplantation (HSCT) is currently treated with systemic corticosteroids despite poor efficacy and side effects. This study investigated the therapeutic effect of budesonide/formoterol, montelukast and n-acetylcysteine, which are suggested as treatment options for BOS after HSCT.

**Methods:**

After diagnosis of BOS, 61 patients were treated with budesonide/formoterol, montelukast and n-acetylcysteine for 3 months. Pulmonary function test and COPD assessment test (CAT) were performed before and after the combination therapy. Therapeutic response was evaluated by changes in forced expiratory volume in 1 s (FEV_1_) or CAT score.

**Results:**

After 3 months of combination treatment, mean FEV_1_ increased by 220 mL (*p* < 0.001) and residual volume decreased by 200 mL (*p* =0 .005). Median CAT score also significantly decreased from 15.5 to 11.0 (*p* = 0.001). The overall response rate to combination therapy was 82 %. Comparing the no-response group and the response group, the forced vital capacity (% predicted) decline between pre-HSCT and BOS diagnosis was significantly greater in the response group (*p* = 0.036).

**Conclusion:**

Combination treatment with budesonide/formoterol, montelukast and n-acetylcysteine significantly improved lung function and respiratory symptoms in patients with BOS after allogeneic HSCT without serious side effects.

## Background

Bronchiolitis obliterans syndrome (BOS) is a non-infectious pulmonary complication of hematopoietic stem cell transplantation (HSCT) that results in progressive circumferential fibrosis of the small terminal airways, manifesting as a fixed new-onset airflow obstruction [1, 2]. Although BOS was previously regarded as rare, recent data has reported that the overall prevalence of BOS is 5.5 % in patients receiving allogeneic HSCT and 14 % among patients who develop chronic graft versus host disease (GVHD) [2]. In addition, patients with BOS have poor prognosis, with an overall survival rate of 44 % at 2 years and 13 % at 5 years [3].

The current treatment of post-HSCT BOS mainly relies on systemic corticosteroids despite their poor efficacy and significant side effects [4]. As a result, several other immunosuppressive and immune-modulating treatments have been investigated as treatment for post-HSCT BOS [5–8]. Recently, potentially less toxic treatments have emerged [4, 8, 9]. Norman et al. retrospectively reviewed that combination therapy of fluticasone, azithromycin and montelukast reduced total corticosteroid exposure in eight HSCT patients with BOS [8]. From prospective studies, montelukast and low-dose macrolide also showed efficacy in BOS after lung transplantation [10, 11]. Bergeron et al. conducted a randomized controlled trial and showed that inhaled budesonide/formoterol led to significant improvement in FEV_1_ in 32 HSCT patients with mild to severe BOS [9]. Despite studies with less toxic treatments showing promising results, more prospective studies with a large study population are required.

In this study, we investigated the impact of combination therapy with budesonide/formoterol, montelukast and n-acetylcysteine in 61 patients with BOS after HSCT. N-acetylcysteine was included based on its potential therapeutic role and low toxicity [12, 13].

## Methods

### Patients

Post-HSCT patients with respiratory symptoms or pulmonary function decline were referred to the pulmonology department from the BMT Center in Seoul St. Mary’s Hospital, Seoul, Korea. After clinical diagnosis of BOS, one experienced pulmonologist (Rhee CK) treated all patients according to the same protocol and follow-up evaluation. After a retrospective chart review, patients treated with budesonide/formoterol, montelukast and n-acetylcysteine during the period between January 2011 and June 2015 were enrolled.

The inclusion criteria were 1) chronic GVHD in other organs and positive diagnostic pulmonary function test (PFT) using the modified NIH criteria [1, 2, 4] and 2) treatment with budesonide/formoterol, montelukast and n-acetylcysteine for at least 3 months. The exclusion criteria were 1) other pulmonary or infectious diseases, such as asthma, lung cancer, COPD, pneumonia or tuberculosis destroyed lungs, and 2) a history of using other inhalers. Approval was obtained from the institutional review board of Seoul St. Mary’s Hospital (KC15RISI0584). The requirement for informed consent was waived by the ethical review board.

### Definition of bronchiolitis obliterans syndrome (BOS)

The diagnostic criteria for BOS were as follows: (1) In patients that underwent a lung biopsy, fibrogenic deposition in the small airways or the bronchioles satisfied the diagnostic criteria for BOS. (2) In patients who did not undergo lung biopsy, chronic GVHD in other organs, air trapping on high-resolution computed tomography (HRCT) and positive diagnostic PFT of the modified NIH criteria [1, 2, 4] were required: (i) forced expiratory volume in 1 s (FEV_1_) of < 75 % of predicted or decrease of the FEV_1_ by 10 % in comparison to the pretransplant value, (ii) FEV_1_/forced vital capacity (FVC) of < 70 % or residual volume (RV) of > 120 % predicted. The reading of HRCT was performed by one radiology specialist (Jung JI [14–16]). During the analysis of each CT examination, the inspiratory images were reviewed before the expiratory images. Air trapping on HRCT was considered as present on the expiratory images when lung regions failed to increase in attenuation and/or failed to decrease in volume compared with the inspiratory images [17]. Patients who satisfied the diagnostic criteria but had not developed an active infectious disease were categorized as having BOS.

### Combination therapy

After diagnosis of BOS, the patients enrolled in this study were treated with a combination of budesonide/formoterol, montelukast and n-acetylcysteine. Patients received 160 μg of budesonide plus 4.5 μg of formoterol fumarate in a dry powder inhaler (Symbicort Turbuhaler; AstraZeneca, Mölndal, Sweden) twice daily, 10 mg montelukast orally daily and 200 mg n-acetylcysteine orally three times a day.

### Pulmonary function test (PFT)

PFT was performed at pre-HSCT, BOS diagnosis and after 3 months of combination therapy. Patients underwent a lung function assessment using a body box plethysmography (SensorMedics Vmax series 22, VIASYS Healthcare, Yorba Linda, CA) to measure flow rates, lung volumes and diffusion capacity.

### COPD assessment test (CAT) scoring

The CAT is a validated self-administered questionnaire that measures health-related quality of life [18]. CAT scores correlate with St. George’s Respiratory Questionnaire scores [18]. The Korean version of the CAT has also been validated [19]. The CAT comprises eight symptoms: cough, phlegm, chest tightness, breathlessness going up hills/stairs, activity limitation at home, confidence leaving home, sleep, and energy [20]. The score of each questionnaire ranges from 0 to 5. CAT scoring was evaluated at BOS diagnosis and after 3 months of treatment.

### Defining the group responding to the combination therapy

After 3 months of combination therapy and following clinical evaluation with PFT and CAT scoring, patients were categorized into two groups according to therapeutic response: above or below the minimum clinically important difference (MCID, the threshold distinguishing between a small meaningless change and a small but meaningful change) [21]. The established MCID for FEV_1_ and CAT are 100 mL [22] and 2 points, respectively [23]. Therapeutic response group was defined as an increase of FEV_1_ more than 100 mL or a decrease in the CAT score of greater than 2 points.

### Statistical analysis

Means and standard deviation were computed for normally distributed continuous variables, whereas medians and interquartile ranges (25th–75th) were used for non-normally distributed continuous data. Categorical data are described as numbers and percentages (%).

For comparison of PFT and CAT scores between BOS diagnosis and after combination treatment for 3 months, the paired *t*-test and the Wilcoxon signed-rank test were performed, respectively. For comparison of continuous variables between the therapeutic response group and the no-response group, student’s *t*-test was performed for normally distributed data and the Mann–Whitney *U* test was used for non-normally distributed data. Categorical variables were compared using the Chi-square and the Fisher’s exact tests as appropriate. Missing values were excluded from the analysis. Statistical analysis was performed using SPSS version 18.0 (SPSS Inc., Chicago, IL, USA). A value of *p* < 0.05 was considered statistically significant.

## Results

### Baseline characteristics of all patients with BOS

Of the 165 patients who were diagnosed with BOS in the pulmonology department from January 2011 to June 2015, 61 patients met the inclusion criteria. Patients’ basic characteristics are shown in Table 1. All patients received combination therapy (budesonide/formoterol, montelukast and n-acetylcysteine) for at least 3 months without serious side effects. Oral candidiasis was reported in four patients and nausea was observed in one patient; these minor problems were well controlled with appropriate support. Patients showed good compliance to the combination treatment. Among the 61 treated patients, four had a problem with the proper inhaler device use and one had a problem with taking the oral drugs regularly. In our center, all BOS patients are managed by one experienced specialist (Rhee CK). Proper education were provided by special pharmacist and inhaler technique was checked in each visit. Even though, four patients had a problem with the appropriate inhaler technique. Reeducation was performed to improve compliance.

**Table 1:** Basic characteristics of total patients with Bronchiolitis Obliterans Syndrome (BOS)

Characteristics	(*N* = 61)
Recipient sex, male	33/61 (54.1 %)
Recipient age	46.5 ± 12.3
Donor sex, male	32/59 (54.2 %)
Donor age^a^	37.1 ± 11.7
Hematologic malignancy	
AML	17 (27.9 %)
ALL	20 (32.8 %)
CML	1 (1.6 %)
NHL	2 (3.3 %)
MDS	21 (34.4 %)
Donor type	
Unrelated	28 (45.9 %)
Sibling	25 (41.0 %)
FMT	8 (13.1 %)
HLA	
full-match	43 (70.5 %)
mismatch	18 (29.5 %)
Stem cell source	
PB	51 (83.6 %)
BM	8 (13.1 %)
Cord	2 (3.3 %)
Time from HSCT to BOS diagnosis, days	434.0 (275.0–835.5)
Acute GVHD	39 (63.9 %)
Chronic GVHD (except lung)	61 (100.0 %)
Skin	31 (50.8 %)
Oral	42 (68.9 %)
Eyes	36 (59.0 %)
Liver	10 (16.4 %)
Joint	3 (4.9 %)
Maximal score of chronic GVHD (except lung)^b^	1 (1–2)
Systemic steroid use	33 (54.1 %)
Steroid dose, mg (equivalent dose of prednisolone)	2.5 (0.0–15.0)
Tacrolimus	18 (29.5 %)
Cyclosporin	9 (14.8 %)
Mycophenolate mofetil	18 (29.5 %)

^a^Age of two donors were missed because of cord blood transplantation (*N* = 59). ^b^Each organ was scored as 0, 1, 2 or 3 based on the degree of functional impairment. Data represent the mean ± SD, median (IQR) or n (%). *BOS* bronchiolitis obliterans syndrome, *AML* acute myeloid leukemia, *ALL* acute lymphoblastic leukemia, *CML* chronic myelogenous leukemia, *NHL* non-Hodgkin lymphoma, *MDS* myelodysplastic syndrome, *FMT* familial-mismatched/haploidentical transplantation, *HLA* human leukocyte antigen, *PB* peripheral blood, *BM* bone marrow, *HSCT* hematopoietic stem cell transplantation, *GVHD* graft-versus-host disease

### Change in pulmonary function after 3 months combination therapy

**Fig. 1:**
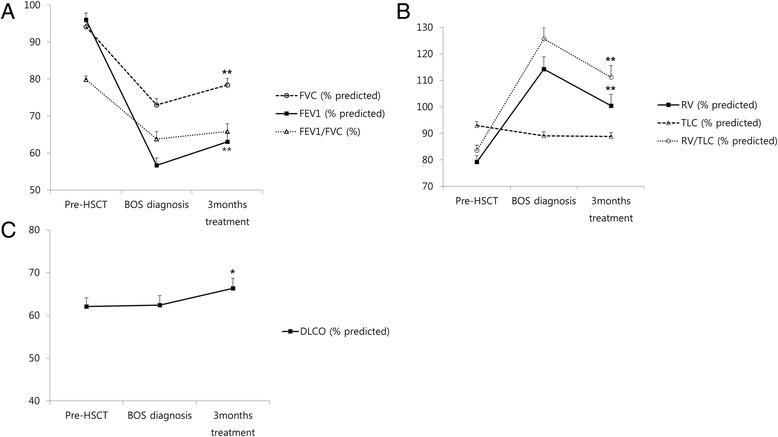
Changes in pulmonary function after 3 months combination therapy. **a** After 3 months of combination treatment, FEV_1_ (% predicted) and FVC (% predicted) increased significantly. Percentage of FEV_1_ and FVC also increased after combination therapy, but the results were not significant. **b** RV (% predicted) and RV/TLC (% predicted) significantly decreased with combination therapy, whereas TLC (% predicted) did not change. **c** DLCO significantly improved with combination therapy. *p < 0.05, **p < 0.01 compared with measurements at BOS diagnosis

Table 2 and Fig. 1 show pulmonary function at pre-HSCT, BOS diagnosis and 3 months after treatment. After treatment, FEV_1_ (L) and FVC (L) increased significantly compared to measurements at BOS diagnosis (0.22 ± 0.43 L and 0.23 ± 0.43 L, respectively; *p <* 0.001, for both). Percentage of FEV_1_ and FVC also increased significantly. Although statistically borderline, FEV_1_/FVC increased with combination treatment (63.80 ± 15.34 at BOS diagnosis and 65.83 ± 16.25 after 3 months treatment; *p =* 0.054). RV (L) and RV/TLC (%) significantly decreased with the therapy (*p =* 0.005 and *p <* 0.001, respectively). The ratio of RV/TLC to predicted value also significantly decreased (*p <* 0.001). There was no significant change in TLC with combination treatment. DLCO improved with combination therapy (*p =* 0.027).

**Table 2:** Changes of pulmonary function test after 3 months combination therapy

Clinical variable	Pre-HSCT^a^	BOS diagnosis	3 months treatment	Change of lung function between 3 months treatment and BOS diagnosis	*P-*value (BOS diagnosis vs 3 months treatment)
FVC (L)	3.70 ± 0.86	2.95 ± 0.81	3.18 ± 0.81	0.23 ± 0.43	**< .001**
FVC (% predicted)	94.14 ± 12.71	73.02 ± 13.23	78.41 ± 13.87	5.39 ± 10.11	**< .001**
FEV1 (L)	2.94 ± 0.69	1.85 ± 0.57	2.07 ± 0.68	0.22 ± 0.43	**< .001**
FEV1 (% predicted)	96.00 ± 13.68	56.68 ± 15.48	63.03 ± 19.31	6.36 ± 12.72	**< .001**
FEV1/FVC (%)	79.86 ± 7.14	63.80 ± 15.34	65.83 ± 16.25	2.04 ± 8.09	.054
RV(L)	1.37 ± 0.38	2.04 ± 0.70	1.84 ± 0.68	−0.20 ± 0.47	**.005**
RV (% predicted)	79.23 ± 17.64	114.24 ± 36.48	100.41 ± 34.06	−12.90 ± 25.71	**.005**
TLC (L)	5.11 ± 1.06	5.03 ± 1.13	5.07 ± 1.07	0.04 ± 0.46	.510
TLC (% predicted)	92.93 ± 10.80	89.04 ± 12.31	88.82 ± 11.09	0.45 ± 7.65	.675
RV/TLC (%)	27.02 ± 5.65	40.35 ± 9.79	36.12 ± 10.28	−4.35 ± 7.34	**< .001**
RV/TLC (% predicted)	83.50 ± 15.77	125.71 ± 33.70	111.22 ± 33.98	−14.06 ± 24.22	**< .001**
DLCO (% predicted)	62.05 ± 15.51	62.39 ± 17.46	66.32 ± 18.38	4.41 ± 13.80	**.027**

^a^Four missing values in Pre-HSCT (*N* = 57). Data represent the mean ± SD. *P*-values shown in bold are significant at the 0.05 level. *HSCT* hematopoietic stem cell transplantation, *BOS* bronchiolitis obliterans syndrome, *FVC* forced vital capacity, *FEV1* forced expiratory volume in 1s, *RV* residual volume, *TLC* total lung capacity, *DLCO* carbon monoxide diffusion in the lung

### Change in CAT score after combination therapy

Fifty-two patients completed the CAT questionnaire at the time of BOS diagnosis and after finishing 3 months combination treatment (Table 3). After treatment, the total CAT score decreased significantly (*p = 0.001*). Total CAT scores at BOS diagnosis and after 3 months treatment were 15.5 (IQR, 9.25 to 19.0) and 11.0 (IQR, 6.0 to 15.75), respectively. When comparing in detail, scores for all individual questions decreased significantly, except for question 2 (phlegm) and question 5 (activities at home).

**Table 3:** Changes of COPD assessment test (CAT) score after therapy (*N* = 52)

Clinical variable	BOS diagnosis	3 months treatment	*P-*value
Q1. Cough	1.5 (0–2)	1 (0–2)	**.022**
Q2. Phlegm	1 (0–2)	1 (0–2)	.983
Q3. Chest tightness	2 (1–3)	1 (0–2)	**.000**
Q4. Breathlessness going up hills/stairs	3 (2–4)	3 (1.25-3)	**.002**
Q5. Activity limitation at home	1 (0–2)	0 (0–1)	.054
Q6. Confidence leaving home	1 (0–3)	1 (0–2)	**.013**
Q7. Sleep	2 (1–3)	1 (0–2.75)	**.004**
Q8. Energy	2 (2–3)	2 (1–3)	**.035**
Total sum of the eight items	15.5 (9.25–19.0)	11.0 (6.0–15.75)	**.001**

Data represent the median (IQR). *P*-values shown in bold are significant at the 0.05 level

### Therapeutic response and association with pulmonary function change

**Fig. 2:**
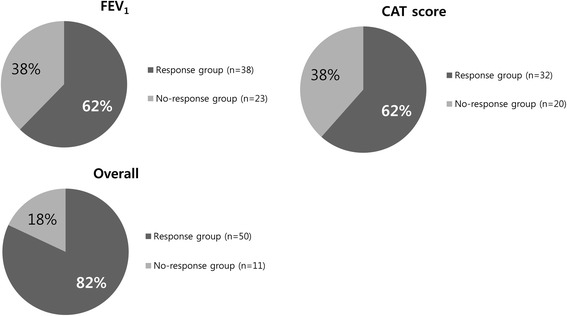
Rate of therapeutic response to combination therapy evaluated by improvement of FEV_1_ or CAT score. Sixty-two percent of patients showed an increase in FEV_1_ greater than 100 mL and the same proportion had a decrease in CAT score of greater than 2 points. When the results of FEV_1_ and CAT score were combined, the overall response rate of the combination therapy was 82 %

Therapeutic response was evaluated by considering the improvement of FEV_1_ or CAT score. Sixty-two percent of patients had an increase in FEV_1_ of greater than 100 mL (Fig. 2). Using the CAT score, 62 % of patients also showed a decrease of greater than 2 points. When the FEV_1_ and CAT score were combined, the overall response rate to combination therapy was 82 %. Comparing the no-response group and the response group, there was no significant difference in baseline characteristics or total CAT score at enrollment (Table 4). PFT at BOS diagnosis also did not significantly differ according to therapeutic response (Table 5). However, the decline of FVC (% predicted) between the time of pre-HSCT and BOS diagnosis was significantly greater in the response group (*p =* 0.036).

**Table 4:** Comparison between therapeutic response group and no-response group

	No-response group	Response group	*P-*value
Recipient sex, male	8/11	25/50	.171
Recipient age	43.6 ± 15.4	47.1 ± 11.7	.389
Donor sex, male	5/11	27/48	.517
Donor age	37.1 ± 9.0	37.1 ± 12.3	.994
Hematologic malignancy			.976
AML	3	14	
ALL	4	16	
CML	0	1	
NHL	0	2	
MDS	4	17	
Donor type			.167
Unrelated	3	25	
Sibling	5	20	
FMT	3	5	
HLA			.717
full-match	7	36	
mismatch	4	14	
Stem cell source			1.000
PB	10	41	
BM	1	7	
Cord	0	2	
Time from HSCT to BOS diagnosis, days	407.0 (272.0–1533.0)	466.0 (274.3–833.3)	.910
Acute GVHD	8/11	31/50	.731
Chronic GVHD (except lung)			
Skin	6/11	25/50	0.785
Oral	7/11	35/50	0.726
Eyes	9/11	27/50	0.106
Liver	2/11	8/50	1.000
Joint	0/11	3/50	1.000
Maximal score of chronic GVHD (except lung)^a^	1 (1–2)	1 (1–2)	0.227
Systemic steroid	8/11	25/50	.171
Steroid dose, mg (equivalent dose of prednisolone)	12.5 (4.4–16.3)	2.5 (0.0–15.0)	.151
Tacrolimus	3/11	15/50	1.000
Cyclosporin	1/11	8/50	.683
Mycophenolate mofetil	5/11	13/50	.275
total CAT score, enroll	10.5 (6.8–16.0)	16.0 (10.0–20.0)	.083

^a^Each organ was scored as 0, 1, 2 or 3 based on the degree of functional impairment. Data represent the mean ± SD, median (IQR) or n. *AML* acute myeloid leukemia, *ALL* acute lymphoblastic leukemia, *CML* chronic myelogenous leukemia, *NHL* non-Hodgkin lymphoma, *MDS* myelodysplastic syndrome, *FMT* familial**-**mismatched/haploidentical transplantation, *HLA* human leukocyte antigen, *PB* peripheral blood, *BM* bone marrow, *HSCT* hematopoietic stem cell transplantation, *BOS* bronchiolitis obliterans syndrome, *GVHD* graft-versus-host disease, *CAT* COPD assessment test

**Table 5:** Association of therapeutic response and pulmonary function change

	No-response group	Response group	*P-*value
BOS diagnosis			
FVC (% predicted)	72.27 ± 15.42	73.19 ± 12.87	.838
FEV1 (% predicted)	56.46 ± 16.05	56.73 ± 15.52	.959
FEV1/FVC (%)	63.64 ± 10.54	63.83 ± 16.29	.970
RV (% predicted)	103.50 ± 39.10	116.68 ± 35.88	.307
TLC (% predicted)	82.30 ± 11.58	90.57 ± 12.07	.054
RV/TLC (%)	37.80 ± 10.76	40.93 ± 9.59	.366
DLCO (% predicted)	58.40 ± 21.81	63.30 ± 16.49	.429
Pre-HSCT – BOS diagnosis^a^			
Δ FVC (% predicted)	12.45 ± 13.28	23.59 ± 15.88	**.036**
Δ FEV1 (% predicted)	30.81 ± 18.16	40.61 ± 18.89	.126
Δ FEV1/FVC (%)	18.18 ± 12.78	14.46 ± 15.63	.467
Δ RV (% predicted)	−20.20 ± 32.45	−36.63 ± 35.90	.194
Δ TLC (% predicted)	3.90 ± 13.85	3.63 ± 11.25	.948
Δ RV/TLC (%)	−9.90 ± 7.69	−14.08 ± 10.17	.232
Δ DLCO (% predicted)	−1.00 ± 20.75	−0.51 ± 16.73	.937

^a^Four missing values in Pre-HSCT (*N* = 57). Data represent the mean ± SD. *P-*values shown in bold are significant at the 0.05 level. *HSCT* hematopoietic stem cell transplantation, *BOS* bronchiolitis obliterans syndrome, *FVC* forced vital capacity, *FEV1* forced expiratory volume in 1s, *RV* residual volume, *TLC* total lung capacity, *DLCO* carbon monoxide diffusion in the lung

## Discussion

In this study, the therapeutic effect of budesonide/formoterol, montelukast and n-acetylcysteine was analyzed in patients with BOS after allogeneic HSCT. After 3 months of treatment, the lung function and respiratory symptoms were significantly improved without significant adverse effects. In addition, the overall response rate to combination therapy was 82 %.

For patients with BOS, the main treatment at present is immunosuppressive agents such as corticosteroids, calcineurin inhibitors, sirolimus, azathioprine, and antithymocyte globulin (ATG) [3, 4]. However, less than 20 % of patients improve and 65 % of patients with BOS will die within 3 years of diagnosis regardless of the therapies administered [1, 24, 25]. Side effects from the immunosuppressive agents are also a problem [4, 24].

Recently, studies with potentially less toxic treatments such as low-dose macrolide antibiotics, leukotriene receptor antagonists, and combinations of inhaled bronchodilators and glucocorticoids have been shown to lead to PFT stabilization or improvement [9–11, 26]. Moreover, a combination of these alternative treatments is under investigation [8, 27, 28]. The rationale for budesonide/formoterol, montelukast and n-acetylcysteine combination therapy, used in our study, is also based on previous reports of each drug.

Inhaled corticosteroids (ICS) were suggested to have therapeutic efficacy and reduce the side effects of systemic treatment in patients with bronchiolitis obliterans (BO) [29]. From a randomized controlled trial, Bergeron et al. reported an improvement in FEV_1_ with budesonide/formoterol combination therapy in patients with BO [7]. The effect of montelukast, a leukotriene receptor antagonist (LTRA), was investigated in other studies. Cysteinyl leukotrienes are known to have important bronchoconstrictive and proinflammatory effects [30]. From prospective studies, Verleden et al. reported adding montelukast as a treatment in patients with BOS [10] and Or et al. showed that montelukast had efficacy in chronic GVHD when added to standard immunosuppressive regimens [31]. Moreover, adding montelukast is a cheap and relatively safe option. Combination of inhaled fluticasone, azithromycin and montelukast was also suggested to halt pulmonary decline and permit reductions in systemic steroid exposure [28]. N-acetylcysteine was suggested to improve clinical conditions and spirometric findings in BOS from randomized clinical trial [13]. N-acetylcysteine is categorized as a mucolytic, but also has antioxidant effects [32]. Reactive oxygen species have also been suggested to play an important role in functional and structural changes in BOS [33]. In an in vitro study using human airway smooth muscle cells, n-acetylcysteine inhibited interleukin (IL)-17 induced IL-8 production, which is highly correlated with BO [33–35].

Although BOS had been thought as irreversible lung disease and most studies focused on disease stability rather than an improvement in lung function [10, 28, 29], our results showed a significant improvement in lung function and symptoms. Beneficial effects shown in our combination therapy may depend on bronchodilation, anti-inflammatory and anti-fibrotic effects. The precise mechanism, interaction and beneficial potency of each drug requires further investigation. Barisone et al. suggested that the airway smooth muscle tone plays a significant role in BOS after HSCT and reversibility [36]. In addition, beneficial effects with FEV_1_ improvement were reported with budesonide/formoterol, azithromycin and N-acetylcysteine treatment, respectively [9, 11, 13]. Previous reports in patients with other obstructive lung diseases can also be used as a reference. The combination of ICS and long-acting bronchodilators (LABA) instead of ICS alone has been suggested to provide synergistic effects on bronchodilation and anti-inflammation [37, 38]. The ICS/LABA combination is also used in severe COPD to reduce exacerbations and improve health status and FEV_1_ compared to mono-therapy [39]. Keith et al. reported the effectiveness of montelukast add-on therapy for managing asthma and allergic rhinitis symptoms [40]. From a meta-analysis, LTRAs as a monotherapy improved asthma control compared with placebo [41]. The addition of n-acetylcysteine in COPD further improved respiratory symptoms, whereas there was no significant change in FEV_1_ [42, 43]. Long-term use of n-acetylcysteine has shown benefits in the prevention of COPD exacerbation [44].

As a result, we suggest that the PFT improvement mainly originated from ICS/LABA inhalation. The addition of montelukast and n-acetylcysteine may contribute to improvements in respiratory symptoms. This suggestion coincides with results from Bergeron et al. [9], who used budesonide/formoterol for BOS after HSCT and observed a significant improvement in FEV_1_, though no changes were seen in respiratory symptoms. However, this suggestion should be proved by well-designed clinical trials in the future.

In this study, the decline in FVC between pre-HSCT and BOS diagnosis was significantly greater in the response group compared to the non-response group. However, this finding does not imply that the combination regimen in our study is effective only in advanced patients. A good response in patients with a significantly declined FVC may result from low lung function. Further studies are required to identify determinants of good response to the combination therapy. In addition, PFT before transplantation is important as a reference for later PFT measurements and helps early diagnosis [45]. Early diagnosis and treatment of BO may improve response over therapy initiated after structural, fibrotic changes have occurred.

We are aware of limitations in this study. Although we employed a consistent treatment plan and regular follow-up by an expert pulmonologist, this study was not a randomized controlled study and the effect of the combination therapy was not compared with a placebo group. Second, the beneficial effects of our combination therapy in lung function and respiratory symptoms require further investigation assessing each drug and their interactions. It is unclear that the improved lung function or symptoms were from the budesonide/formoterol inhalation or the combination treatment. Third, further studies are required to assess long-term outcome and survival benefits. Fourth, analyzing the effect of the combination treatment considering the changes of other systemic immunosuppressive medications were not done in this study. Finally, patients in our study received education about the appropriate use of budesonide/formoterol Turbuhaler and showed good compliance. However, more precise measurements using the inhaler and studies with other inhalers and devices are also required.

## Conclusion

The combination of budesonide/formoterol, montelukast and n-acetylcysteine significantly improved lung function and respiratory symptoms in patients with BOS after allogeneic HSCT. Furthermore, combination therapy showed better therapeutic response in patients with BOS who showed prominent lung function decreases between pre-HSCT and BOS diagnosis.
